# Annotations

**Published:** 1888-10-20

**Authors:** 


### Annotations.

Sir Morell Mackenzie's Defence.

Wrangling and fighting seem to be the normal condition of mind in our nineteenth-century life. When politicians fight, the personal element is more or less obscured, and principles at least appear to be pushed to the front. It is more than probable, however, that even in the political world principles occupy a secondary place as actual causes of quarrel, whilst personal misunderstandings, jealousies, and enmities are the really efficient agents. Doctors are proverbial for their quarrelsomeness. So much is this the case that a doctors' wrangle ordinarily excites nothing more than good-natured contempt in the spectator. The last quarrel in the medical world has had an origin without parallel, and has attained a magnitude which has never before been witnessed. It is not too much to say that all Europe and America are interested in the combat. The daily papers have furnished fairly full accounts of the struggle, and some of them have commented with much acuteness on the matter offered for their consideration. Sir Morell Mackenzie puts his case with skill, and succeeds in showing that whatever his own personal behaviour may have been, one at least of his German colleagues did not act with that severe self-repression which the occasion undoubtedly demanded. The points of professional interest can only be decided by experts. The matter, however, is very simple. From Sir Morell's point of view, an uncertain diagnosis necessitated an expectant treatment. The uncertainty of the diagnosis resulted from Professor Virchow's decision that the portion of the growth examined by him did not show any true cancer cells. Taking into consideration the age of the illustrious patient, his comparatively feeble strength, the absence of certainty as to the nature of the case, and the unquestionably tremendous risk involved in the radical operation, it seems to have been the course which most sober people would have recommended, that the Emperor should at least wait before submitting himself to an operation which would almost certainly have ended in death. That, at least, is the view which a dispassionate critic, considering the whole of the evidence, would naturally be inclined to adopt. It is possible that an operation might have prolonged life; might even have resulted in a permanent cure. Possible—but only possible. The probabilities were as fifty to one in favour of a different and fatal result.

Gerhardt and Bergmann's View of the Case.

Those who desire to judge fairly as to the merits of the unprecedented controversy of which the late German Emperor was the innocent cause, will take care to give due weight to the points raised by the German physicians. The world, both lay and medical, has something to learn from the case, and the true lessons can only be learnt by eliminating the question of nationality. Disease, like religion, knows nothing of national differences. Men of education and worldly experience show to small advantage when nationality blinds their eyes to the truth. It must not be supposed that the German physicians have nothing important to say in justification of their own contention. A professional critic pointed out the other day a circumstance which has been pretty generally overlooked in connexion with the case. It is this: That Sir Morell Mackenzie was first called in consultation by the wish of the German doctors. Having "examined" and expressed his opinion as to the nature of the disease and its treatment, it was then his duty, in accordance with the ordinary rules of professional etiquette, to have retired and left the future management of the patient in the hands of his ordinary professional advisers. Instead of that, he not only remained with the sick man, but also had with him an English professional colleague. That was a not unnatural cause of offence to the German doctors. In ordinary circumstances no such course would have been even thought of. Then it must be admitted that, though the German pathologist, Virchow, declared he did not find cancer cells in the portion of the growth examined by him, yet the German physicians professed at an early stage their decided conviction that the case was one of cancer. It is true also, as they assert, and as all the medical world knows, that if an operation for cancer is ever serviceable it is serviceable at the very commencement of the growth. The German physicians are fully entitled to say that they were right as to diagnosis, and they are equally entitled to their opinion that an early operation would have effected a cure, and at any rate ought to have been tried. The case lies in a nutshell. The doctors have disagreed, which, being men, it is not unnatural they should have done. The patient is dead, he and his friends having elected to adopt the course recommended by one doctor. Had the course recommended by the other doctors been followed he might have died sooner than he did, or he might have been alive to-day. Which result would have followed no mortal man can affirm. The moral which is pre-eminently pointed by the story is that all human beings doctors included, are very liable to error, and that kings are subject to the same dangers and the same uncertainties as all other men.

Leeds as a Leader.

We have often had occasion to congratulate Northerners on the great judgment and skill which they bring to bear upon philanthropic enterprises of all kinds. In hospital matters they set the whole country an example which we can only hope will one day be followed by London and the South. Working-men in the North take a pride in ministering to the efficiency and maintenance of the hospitals. They organize collections, and raise annually as much, and sometimes more, money than all the other classes in the town put together. This is as it should be, because it encourages the wealthier residents to bestir themselves when new buildings are required, involving an extraordinary expenditure of thousands of pounds. Rather more than a year ago the artisans of Leeds organised a Hospitals Week, and so attracted the interest and support of the workshops of every degree throughout the town and district. The effect of this movement on the hospital resources was as remarkable as might well be expected. We have now the pleasure to record that the committee of the Leeds Infirmary, finding it necessary to make some considerable extensions to the Infirmary buildings, issued an appeal for £30,000. Within a month's time the whole £30,000 was subscribed, and there would have been no difficulty in obtaining a larger sum if the list had been kept open a few days longer. Anything more creditable to the town of Leeds, or more flattering to the Infirmary Committee and their general manager (Mr. Thomas Blair), it would be difficult to imagine. Would that Londoners could be aroused to take an equal interest in their voluntary hospitals. We should not then have a much-needed hospital like the Great Northern Central left in a half-finished condition for want of £20,000, despite the urgent necessities of the poor in North London, for whose benefit it is maintained and worked. The Great Northern Central is in fact an instance which may be fairly contrasted with that of Leeds, much to the discredit of Northern London. To begin with, the population of the metropolitan district which is to be supplied by this hospital is twice as large as that of Leeds. Then the wealth of North London must be much greater than that of Leeds. Finally, Leeds has raised £30,000, as against £25,000 raised by North London. It has taken North London seven years to raise twenty-five thousand. It has not taken Leeds three weeks. And yet the metropolitan people regard themselves as being in every way superior to the people of country towns. Truly, Londoners do not need to use the Scotchman's prayer: "Lord, gie us a guid conceit o' oorsels."

